# Genome-wide identification of the *ZIP* gene family in lettuce (*Lactuca sativa L*.) and expression analysis under different element stress

**DOI:** 10.1371/journal.pone.0274319

**Published:** 2022-09-28

**Authors:** Feng Gao, Jing Li, Jing Zhang, Nenghui Li, Chaonan Tang, Emily Patience Bakpa, Jianming Xie

**Affiliations:** College of Horticulture, Gansu Agricultural University, Lanzhou, China; University of Tsukuba, JAPAN

## Abstract

The *ZIP* protein (*ZRT*, the *IRT*-like protein) is an important metal transporter that transports Zn, Fe, and other divalent metal ions in plants. In this study, we identified 20 *ZIP* genes in lettuce (*Lactuca sativa L*.). We used bioinformatics methods and renamed them according to their E value in hmmsearch. We also analyzed their gene structure, chromosomal location, constructed a phylogenetic tree, conserved motifs, performed synonymous analysis and responses to abiotic stresses. The results show that these *LsZIP* genes have 3–11 exons and were distributed unequally on 8 of the 9 chromosomes in lettuce. Based on phylogenetic analyses, the *LsZIP* gene family can be divided into three subfamilies, and the *LsZIP* genes within the same subfamily shared similar gene structure. The *LsZIP* genes contain 12 Motifs, of which Motif1 to Motif8 are widely distributed in group Ⅰ. Furthermore, the *LsZIP* gene contains numerous hormones and anti-stress response elements. Real-time quantitative PCR demonstrated that most *LsZIP* genes is up-regulated under the elemental stress in this experiment, indicating that they are positively regulated. But different elemental stressors can induce the expression of *LsZIP* gene to varying degrees. The *LsZIP* genes also change in response to different elemental stresses. The present study serves as a basic foundation for future functional studies on the lettuce *ZIP* family.

## Introduction

The gene family *ZIP* (*ZRT*, the *IRT*-like protein) is an important metal transport protein, found in many organisms, including bacteria, fungi, animals and plants. It contains a variety of metal substrate-specific membrane transport proteins and has been shown to be involved in the transfer and absorption of divalent metal ions by plants [1]. In general, the *ZIP* proteins have similar membrane topology and eight potential transmembrane domains. They are rich in conserved histidine residues and variable region contains potential metal binding domains. There is a variable region between TM-3 and TM-4, and the outer surface of the plasma membrane contains amino- and carboxyl-terminals [2]. *ZIP* genes were first identified in Arabidopsis thaliana in plants.

Several *ZIP* genes have been shown to be expressed in the absence of zinc (Zn) to low levels of Zn ions. Among them are *AtZIP1* and *AtZIP3*, which are expressed in roots. *AtZIP4* transports Zn ions into cells or between plant tissues [3]. Subsequent function verification studies have shown that *ZIP* transporters such as *AtZIP1*, *AtZIP2*, *AtZIP3* and *AtZIP4* are functionally transporters with different metal affinities [4, 5]. Studies have also shown that *ZIP* transporters are directly involved in the accumulation of Zn in edible plant parts [6, 7]. The expression level of *ZIP* family genes in plants shows a dynamic pattern. For example, under conditions of Zn deficiency, some *ZIP* transporters are more highly expressed, but when the Zn levels return to normal or high, their expression is lower. Therefore, when Zn is added to a medium with Zn deficiency levels, their expression decreases within 2 hours [8]. Several *ZIP* genes are involved in the uptake and transport of Zn in different plant parts. In rice, overexpression of *OsZIP1* increases the accumulation of Zn and iron in roots, shoots, and seeds [9, 10]. On the contrary, over-expression of *OsZIP4* and *OsZIP5* can significantly increase the Zn concentration in the roots of transgenic plants, but in seeds, the Zn content cannot be increased [11, 12]. Similarly, over-expression of the Zn transporter *TdZIP1* in wild wheat can lead to excessive accumulation of Zn in cells, resulting in Zn toxicity [13]. In general, the transporter *ZIP* is mainly responsible for the uptake and transport of Zn and Fe. However, these proteins may also play a role in the transport of other metals, such as Mn and Cu [14, 15]. In addition, they are also involved in the uptake, transport and accumulation of cadmium (Cd) and various toxic heavy metals in plants [16, 17]. Research shows that the *ZIP* gene is involved in the absorption and transportation of Cd. For example, in Chinese cabbage, *ZIP2* and *ZIP3* were found to be associated with the transport of Cd and showed high expression levels [18]. The expression of *SlZIP4* was positively correlated with Cd concentration in a certain concentration range in tomato [19]. In addition, *ZIP* genes are also found in mulberry, rice, cole and tobacco, which are involved in the uptake and transport of Cd [16, 20–23].

Lettuce (*Lactuca sativa*) is an important vegetable crop originally from the Mediterranean coast and cultivated worldwide [23]. It, is also a source of various minerals, vitamins, and fiber and has a low caloric content [22, 24]. It is usually used as a fresh vegetable in salads. In 2018, the global production of lettuce and chicory was 27,256,487 tons (http://www.fao.org/faostat/en/#data/QC/visualize). Among the cultivated crop species, lettuce is known for higher Cd uptake, and higher accumulation in the leaves. Lettuce has a high rate of Cd uptake and transport. When grown on soil contaminated with Cd, it can quickly transport Cd to the buds [25]. Otherwise, lettuce is very tolerance to Cd. The Cd content in the leaves can exceed 100 times the legal maximum level of commercially available vegetables for human consumption, and the plants shows no symptoms of Cd stress. Lettuce can accumulate Cd not only at high Cd levels [26], but also at low Cd levels [27], so lettuce has always been an important source of dietary Cd uptake by the human body. In the United States, lettuce is the largest contributor to Cd intake in adolescents and adults and the fourth largest contributor to total Cd intake in children [28]. Therefore, genes related to Cd uptake and transport in lettuce are of critical safety and important. In some plant species, the *ZIP* gene has been studied. However, there is no relevant research report on the *ZIP* protein in lettuce. Because of the importance of *ZIP* genes in metal ion uptake and transport and the specificity of lettuce in Cd uptake, it is reasonable to conduct a systematic study of the *ZIP* family in lettuce. With the completion of the genome sequencing of lettuce, we study the evolution of the lettuce *ZIP* gene family at the entire genome level and the expression tissue characteristics [29].

## Materials and methods

### Identification and database source of lettuce *ZIP* gene family

We downloaded the complete genome sequence databases of lettuce from the NCBI database (Genome v7: ID:6720851) and downloaded the hidden Markov model (HMM) file of the *ZIP* domain (PF02535) from the Pfam protein family database (http://pfam.xfam.org/). The *ZIP* protein search was performed using the standard HMMER 3.0 software parameters from the Salad Gene database [30–33]. For the candidate genes obtained from the search, we used the SMART and PFAM programs to confirm that they contained specific domains, and manually removed *ZIP* genes that did not contain domains and duplicates [34, 35]. Finally, we obtained 20 *ZIP* gene models. Then, the ProtParam tool (http://web.expasy.org/protparam/) was used to determine the sequence length, molecular weight, isoelectric point, and instability index of these putative *LsZIP* genes [36]. Finally, the subcellular location of *LsZIP* was determined using Wolf-PSORT (https://wolfpsort.hgc.jp/) [37].

### Analysis of gene structure and conservative motifs of the *LsZIP*s gene

To clarify the evolutionary relationship of 20 *LsZIP* genes, a multiple sequence alignment of 20 *LsZIP* sequences was performed using Clustal X software [38]. The *LsZIP* gene structures were examined using the Gene Structure Display Server (GSDS: http://gsds.gao-lab.org) to map the exon and intron composition. MEME online program (http://meme-suite.org/tools/meme) was used to identify conserved motifs of the lettuce *ZIP* protein with specific domains [39]. The optimized parameters were as follows: the maximum number of motifs was set to 12, the number of repeats was set to arbitrary, and the optimal width of each motif was set between 6 and 50 residues.

### Phylogenetic analysis and classification of lettuce *ZIP* gene family

According to the description in the literature, the *ZIP* protein sequences of 14 species (*Cucumis sativus*, *Arabidopsis thaliana*, *Oryza sativa L*., *Zea mays L*., *Brachypodium distachyum L*., *Setaria Italica L*., *Medicago tribuloides*, *Glycine max Merr*., *Solanum lycopersicum*, *Hordeum vulgare L*., *Brassica juncea*, *Triticum aestivum L*., *Sorghum bicolor L*. and *Panicum hallii*) were downloaded from phytozome (www.phytozome.net). ClustalX was used to perform a multiple sequence alignment of these *ZIP* proteins (default parameters). Their phylogenetic tree was analyzed and constructed using MEGA7.0 software. The phylogenetic tree construction method used the Neighbor-Joining (NJ) method, and 1000 bootstrap replicates were filmed [40]. In contrast, the family phylogenetic tree of the 20 *LsZIP* genes was also constructed using the same method and parameters. Besides, we predicted the secondary structure of lettuce *ZIP* protein using SOPMA software [41], and we also inferred the 3D structure of lettuce *ZIP* protein based on the protein homology model in the swiss model database [42].

### Analysis of cis-acting elements of *LsZIP* genes

To identify the cis-regulatory changes in *LsZIP*s under elemental stress, the PlantCARE Server (http://bioinformatics.psb.ugent.be/webtools/plantcare/html/) was used to extract the sequence 2000 bp upstream of the *LsZIP*s promoter to determind cis-acting elements. The results are visualized using GSDS.

### The distribution of *LsZIP* genes on the chromosome and syntenic analysis

Using the gene positions obtained from the lettuce gene database, 20 *LsZIP*s on 8 lettuce chromosomes were mapped and the Multiple Collinearity Scan toolkit (MCScanX), was used to analyze gene repetitions (default parameters) [43]. Subsequently, MCScanX and Circos were used to visualize syntenic analysis maps. To measure the selection pressure of *LsZIP* genes in evolution, TBTools software was used to calculate the non-synonymous (Ka) and synonymous (Ks) replacement rate of the *LsZIP* gene, and the Ka/Ks ratio was determined [44].

### Plant materials and element stress treatments

According to the results of previous experiments, the Cd-sensitive variety ‘lusu’ lettuce is the cultivar in this experiment [26]. To ensure uniform germination, the seeds were cultured in distilled water for 6 hours, then the swollen seeds were placed in a germination tray to accelerate germination. After 48 hours of germination, selected lettuce seedlings with a root length of 1 cm were planted on a plug tray filled with quartz sand and placed in an artificial climate box with 70% relative humidity, 22±2 °C, and 16 h light/8 h dark, light intensity 200 μmol m^-2^*s^-1^, and irrigated with water. After a growth period of 7 days, they were transferred into a 1 litre hydroponic container containing 1/2 strength of Hoagland nutrient solution (pH = 6). Four, lettuce plants in each hydroponic container were grown for another week until the third leaf appeared, then the seedlings were transferred into a new container for treatment. Treatments were applied at two levels: of Zn-deficiency 0 mg/l and Zn-excess 10 mg/l; two levels: Fe-deficiency 0 mg/l and Fe-excess 200 mg/l; and one level of Cd 10 mg/l. The leaves were harvested after 0 h, 12 h, 24 h and 48 h. Three separate plants per point were used for each stress level, and these samples were quickly frozen in liquid nitrogen and then stored at -80°C.

### Quantitative real-time PCR analyses

SpeadyPure Plant RNA Extraction kit (AG, https://agbio.com.cn) was used for Total RNA. 400 ng total RNA and random primers were added to Evo M-MLV RT Premix for cDNA synthesis (AG, https://agbio.com.cn). qRT-PCR was performed using SYBR Green Pro Taq HS qPCR Kit (AG, https://agbio.com.cn). The online primer design (S1 Table) was carried out through the homepage of the Shanghai Biological Company (Shanghai, China) and submitted to the company for synthesis. Isopentenyl diphosphate isomerase 2 (*IPP2*; Ls2g17540) was used as a reference gene [45]. Three biological replicates were performed for each reaction and the results were analyzed by the 2-ΔΔCT method [46]. The relative fold change was compared to the expression level at 0 h at other time points, and the expression level at 0 h was normalized to 1. The sequences of the PCR primers are listed in S2 Table.

## Results

### Identification of the *ZIP* proteins in lettuce

Using the HMM file of the *ZIP* gene, 22 candidate gene models were searched through the HMMER3.0 program. The HMMER and SMART programs were used to check the annotations of these gene models to ensure that they contained specific domains. After verification, two repeated sequences (PLY96399.1 and PLY94661.1) were manually deleted. Finally, based on the presence of a complete *ZIP* domain on the surface, 20 gene models were selected and labeled as lettuce *ZIP* genes. The total 20 *ZIP* genes are distributed on chromosomes 1–8 and were renamed *LsZIP1* to *LsZIP2*0 according to their match values in HMMER search results. In this work, we identified 20 lettuce *ZIP* genes and divided them into three categories. The physical and chemical properties and subcellular location of the 20 *LsZIP* proteins are listed in S2 Table. Among the 20 *LsZIP* proteins, *LsZIP19* was determined to be the smallest protein with 199 amino acids (aa), and the largest was *LsZIP20* (578 aa). 18 *LsZIP* proteins are classified stable (instability index <40), while *LsZIP*12 and *LsZIP*14 are unstable (instability index> 40). The molecular weight of *LsZIP* protein ranges from 21.48 to 60.51 kDa, and the pI value ranges from 5.31 to 9.4. The predicted subcellular localization results show that 18 *LsZIP* proteins are localized in the Plasma membrane region and the remaining two proteins are located in the vacuole.

### Phylogenetic analyses of the of *LsZIP* and 14 plant *ZIP* proteins

We collected 133 *ZIP* genes encoding protein sequences from 15 species and constructed a phylogenetic tree using the Neighbor Joining (NJ) method to determine their autologous or collateral relationships. As shown in Fig 2, the phylogenetic tree is divided into four clades. The genes of 8 monocot and 7 dicot plants are distributed among four clades. This indicates that the separation of the progenitor genes of the four clades occurred earlier than the separation of monocots and dicots. Among them, clade I and III can be further divided into two subclades, and clade IV can be further divided into four subclades. Except for IVa (contains only dicotyledonous plants) and IVc (contains only monocotyledonous plants), the remaining subclades contain both dicotyledonous plants and monocotyledonous plants. Twelve *AtZIP* genes are distributed among four clades, of which clade I contains two (not included in IB). Clade II contains one, clade III contains three, and clade four contains six (not included in IVc).

*ZIP* genes in lettuce are unevenly distributed among the clade. Clade I has five lettuce *ZIP* genes, clade II has only one gene *LsZIP13*, clade III has five lettuce *ZIP* genes, and clade IV has nine lettuce *ZIP* genes. *ZIP* genes in lettuce are unevenly distributed across the clade. In all clades, the *LsZIP* gene is homologously related to the *AtZIP* gene. With the exception of *LsZIP15* and 16 in Ia, *LsZIP14*, *LsZIP18*, and *LsZIP20* in Ib, which show strong homology with monocots, the remaining lettuce *ZIP* genes resemble dicots. The homology is stronger. In clade IVb, five lettuce *ZIP* genes formed a unique monophyletic group, and *LsZIP5* alone formed a unique monophyletic group. In the smallest clade group, lettuce *ZIP* genes are genetically related to Arabidopsis (*LsZIP2*, *LsZIP4* and *LsZIP6*) and tomato (*LsZIP1*, *LsZIP3*, *LsZIP8*, *LsZIP15*), cucumber (*LsZIP9*, *LsZIP12*), and soybean (*LsZIP13*). More recently, this suggest that they may share a common ancestor. In addition, some *LsZIP* genes are arranged in pairs of smaller evolutionary branches, namely (*LsZIP9/12*, *LsZIP2/4/6*, *LsZIP1/3* and *LsZIP14/18/20*), as well as *LsZIP8*, *LsZIP15* and *LsZIP16* are distributed separately in evolutionary branches. This indicates that the lettuce *ZIP* gene family can spread by tandem duplication, segmental duplication, and transposition events. In the smallest clade, lettuce *ZIP* genes are genetically related to Arabidopsis (*LsZIP2*, *LsZIP4* and *LsZIP6*) and tomato *(LsZIP1*, *LsZIP3*, *LsZIP8*, *LsZIP15*), cucumber (*LsZIP9*, *LsZIP12*) and soybean (*LsZIP13*). This suggest that they may have a common ancestor. To assess similarity information between lettuce *ZIP* proteins and *ZIP* proteins of other species and to comprehend the sequence conservation of *ZIP* proteins over evolution. ClustalX was utilized to analyze the multiple sequence alignment of 133 *ZIP* proteins from 15 species, the results are shown in S1 Fig. The 133 *ZIP* proteins contain a total of seven transmembrane (TM) structural domains. In addition, there is a variable region between TM-3 and TM-4 containing a potential metal-binding domain enriched with histidine residues.

### Gene structure and motif composition of lettuce *ZIP gene* family

**Fig 1:**
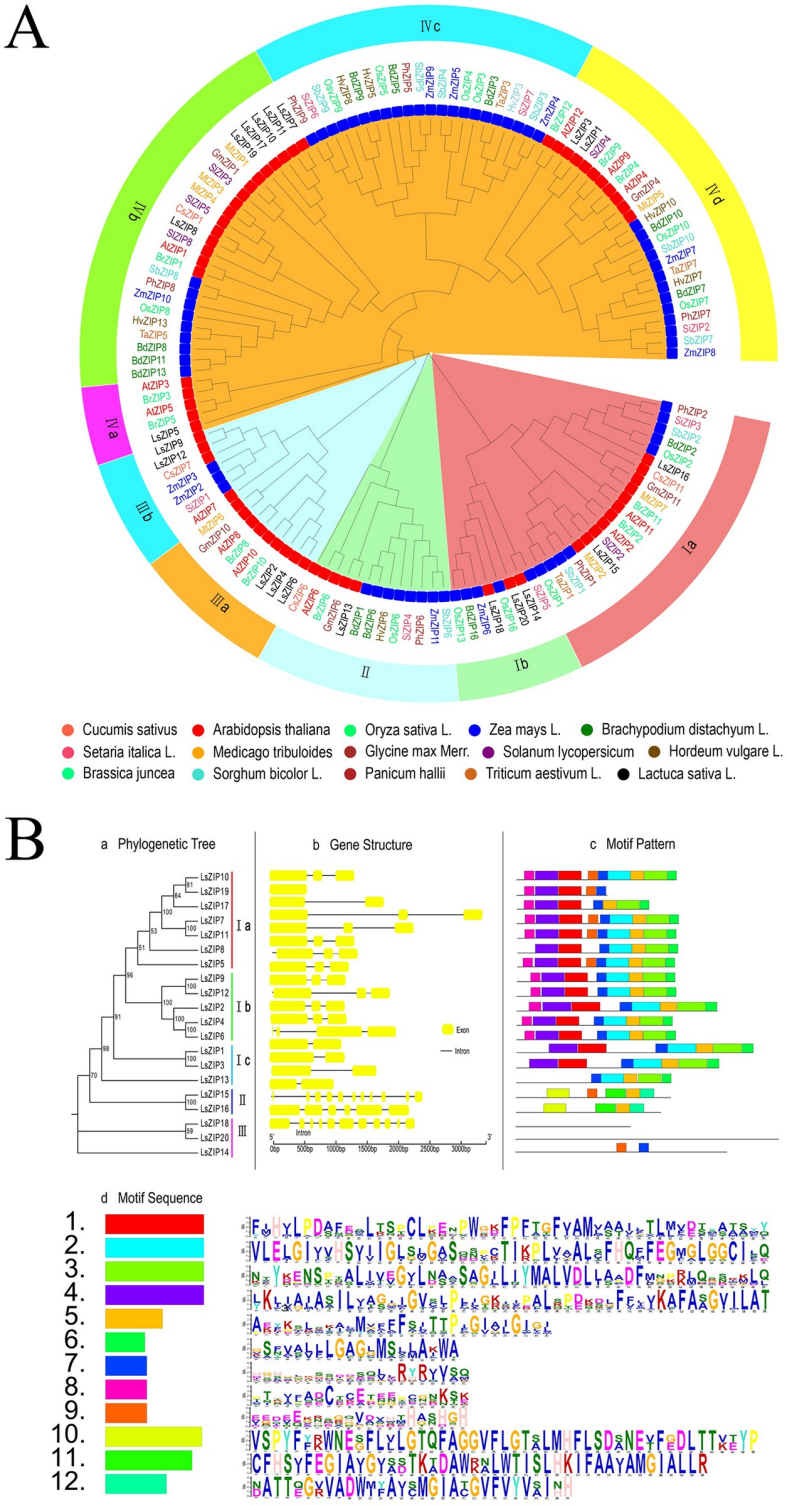
Phylogenetic tree and gene structure analysis of ZIP gene family. **(A)**: phylogenetic analysis of 133 *ZIP* genes from 15 plant species. The phylogenetic tree was constructed using the NJ method. The blue and red rect on the tree indicate Monocot (8) and Dicots (7) species, respectively. The phylogeny tree was visualized by EvolView (https://evolgenius.info//evolview-v2). (B): phylogenetic relationships, gene structure, and architecture of conserved protein motifs in *ZIP* genes from lettuce and the *LsZIP* proteins annotated with the MEME server. (B-a): phylogenetic tree was constructed based on the full-length sequences of lettuce *ZIP* proteins using MEGA 7 Software. Details of clusters are shown in different colors. (B-b): Exon-intron structure of lettuce *ZIP* genes. Yellow boxes indicate exons; black lines indicate introns. (B-c): motif composition of lettuce *ZIP* proteins. Motifs, numbered 1–12 are shown in different colored boxes; the corresponding sequence is shown in B-d.

To gain a deeper understanding of the evolution of the *ZIP* family in lettuce, we mapped the exons-introns of 20 identified *LsZIP* genes. As shown in Fig 1B-a, the twenty *LsZIP* genes are divided into three subfamilies, of which the I family is divided into three small subfamilies. In Fig 1B-b, we see that 17 of the 20 *LsZIP* genes were found to have 1 to 4 exons. *LsZIP20* has 6 exons, *LsZIP14* and *LsZIP18* have 11 exons, while *LsZIP19* has only one exon. In generally, the genes in the same group have similar structures. For example, all members of group Ib contain three exons. Through MEME motif analysis, 12 conserved motifs were found in *LSZIP*. The conserved sequences of the 12 motifs are shown in Fig 1B-d. As shown in Fig 1B-c, motifs 1 to 8 are widely distributed in group I, motifs 10 to 12 are only found in *LsZIP15* and *LsZIP16*, motif 7, motif 9, and motif 12 were found in *LsZIP15*, and no motifs were found in *Lszip16*, *LsZIP18* and *LsZIP20*. In general, *LsZIP* members of the same group have similar motif composition; for example, motif 1 to motif 8 are widely distributed in group I, while motif 10 and motif 11 are specific to group II. The clustered *LsZIP* pairs *LsZIP5/8*, *LsZIP9/12*, and *LsZIP4/6* are even in their motifs. The similar motif arrangement among the *LsZIP* proteins in the subgroup suggests that the protein structure is conserved in a specific subfamily. Generally, *LsZIP* members in the same group have similar conserved motif composition and gene structure, which, together with the results of phylogenetic analysis, indicates that the group classification is reliable.

### Secondary and tertiary structure prediction of *LsZIP* proteins

To determine the function of *LsZIP* proteins, we predicted their secondary and tertiary structures. The results of the secondary structure demonstrated that the percentages of α-helix, β-turn, random coil and extended strandwere 37.19% (*LsZIP19*) to 59.92% (*LsZIP18*), 1.75% (*LsZIP4*) to 7.44% (*LsZIP20*), 10.95% (*LsZIP9*) to 48.24% (*LsZIP19*), and 9.91% (*LsZIP14*) to 36.76% (*LsZIP14*), respectively (S4 Table). The *LsZIP* proteins were dominated by Alpha helix, Random coil and Extended strand.

**Fig 2:**
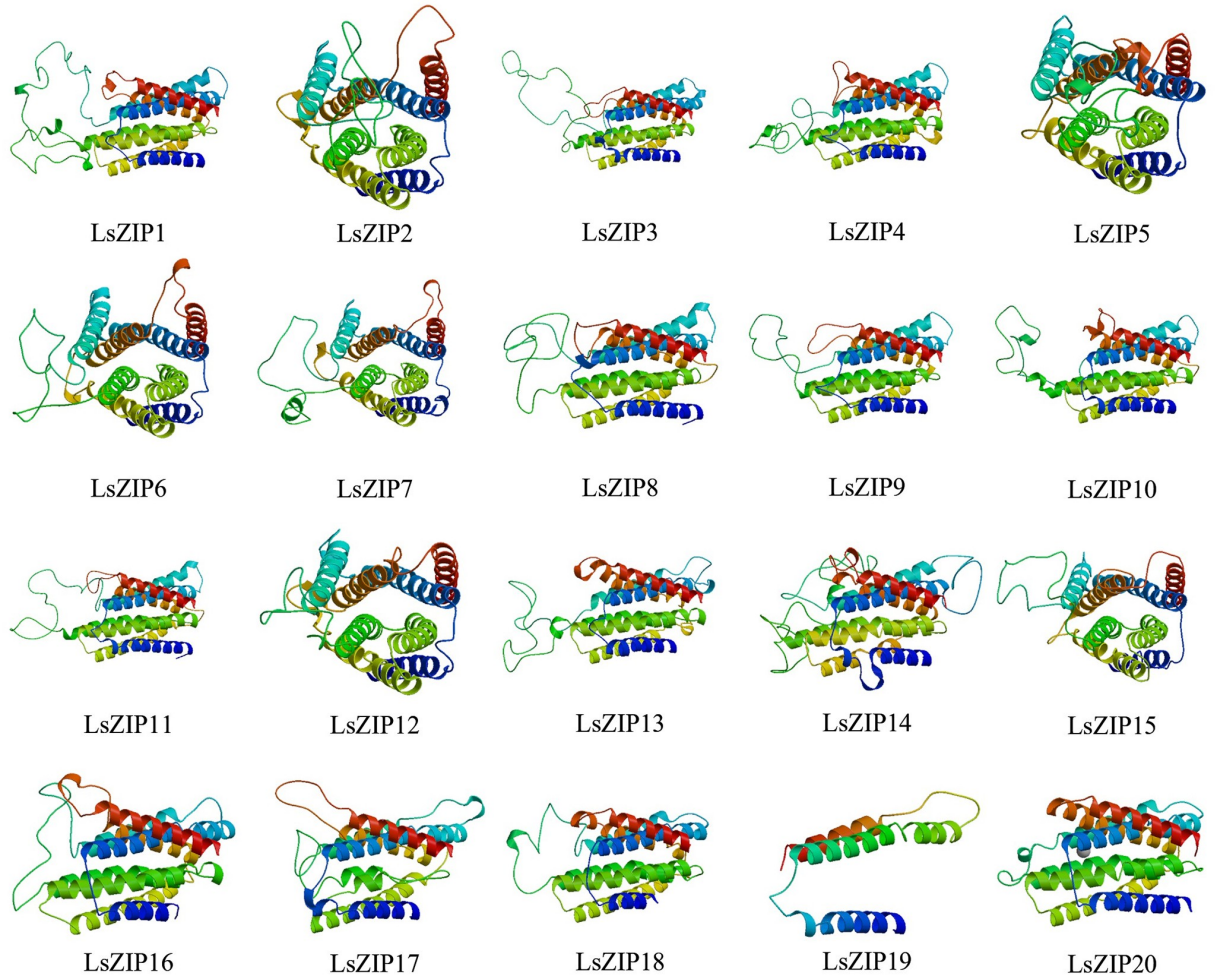
Tertiary structure prediction of 20 *LsZIP* proteins. Using the protein homology modeling method based on the *LsZIP* structure of the swiss-model database, the structure with the highest score was chosen as the optimal structure for the *LsZIP* protein.

The structures of 20 lettuce *ZIP* family member proteins were predicted using a protein homology modeling approach based on lettuce *Zip* structures from the Swiss-model database. The 3D structures of *LsZIP* proteins are shown in Fig 2. The structure with the highest score was selected as the best structure of the *LsZIP* protein. Except for *LsZIP9* (containing only 3 Alpha helix), there was no significant difference in the 3D structures of the *LsZIP* family (containing 8 Alpha helix). Two proteins were identified in all *LsZIP* gene families, including the Bacillus bronchisepticus *ZIP* protein (*BbZIP*) and the membrane protein (Fig 2). This suggests that they may function in the transmembrane transport of Zn. As shown by the simulation analysis, these 20 genes of *LsZIP* have no significant differences at the protein structure level.

### Prediction of cis-acting elements of 20 *LsZIP* genes

**Fig 3:**
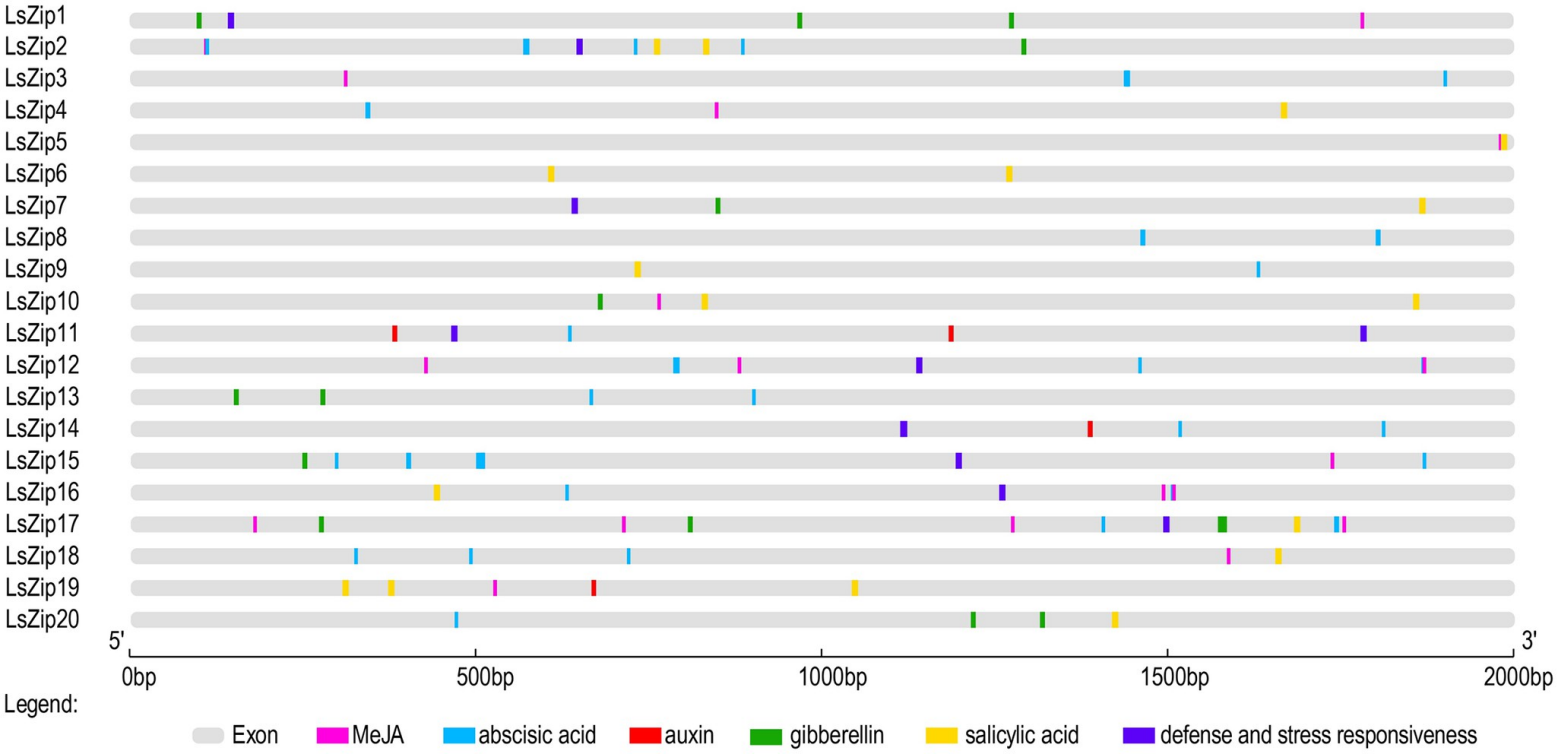
Cis-Elements analysis in putative promoter sequences of *LsZIP*s. Different cis-elements with the same or similar functions are shown in the same shape and color.

To further determine the role of the *LsZIP* gene family in lettuce, we analyzed the possible effects of 20 cis-acting elements in the 2000bp sequence upstream of the *LsZIP* gene promoter. The results showed that the promoter region of the *LsZIP* gene family contains many cis-regulatory elements and plant hormone response elements in response to abiotic stress (Fig 3). The cis-elements are classified into two main subgroups: hormone-responsive and stress-responsive. The cis-resposive regulatory element involved in 39 abscisic acid responses (TACGTGTC、GCAACGTGTC、GACACGTGGC、CGCACGTGTC、CACGTG、ACGTG and AACCCGG) and 17 salicylic acid responses (TCAGAAGAGG and CCATCTTTTT) occurred in 14 and 12 *LsZIP* gene promoter sequences, respectively. Four auxin-responsive cis-regulatory elements (GGTCCAT、AACGAC and TGACGTAA) are distributed in only four *LsZIP* genes, but 14 gibberellin-responsive cis-acting elements (CCTTTTG、TATCCCA and TATCCCA) are distributed in eight *LsZIP* genes. This is somewhat surprising because Zn is an essential element in the synthesis of tryptophan, and tryptophan is the precursor auxin synthesis. 36 MeJA-responsive cis-regulatory elements (TGACG and CGTCA) are distributed among 12 *LsZIP* genes, and 10 defense and stress-responsive cis-regulatory elements are distributed among 9 *LsZIP* genes.

### Distribution of 20 *LsZIP* genes on chromosomes of lettuce and synonymy analysis

**Fig 4:**
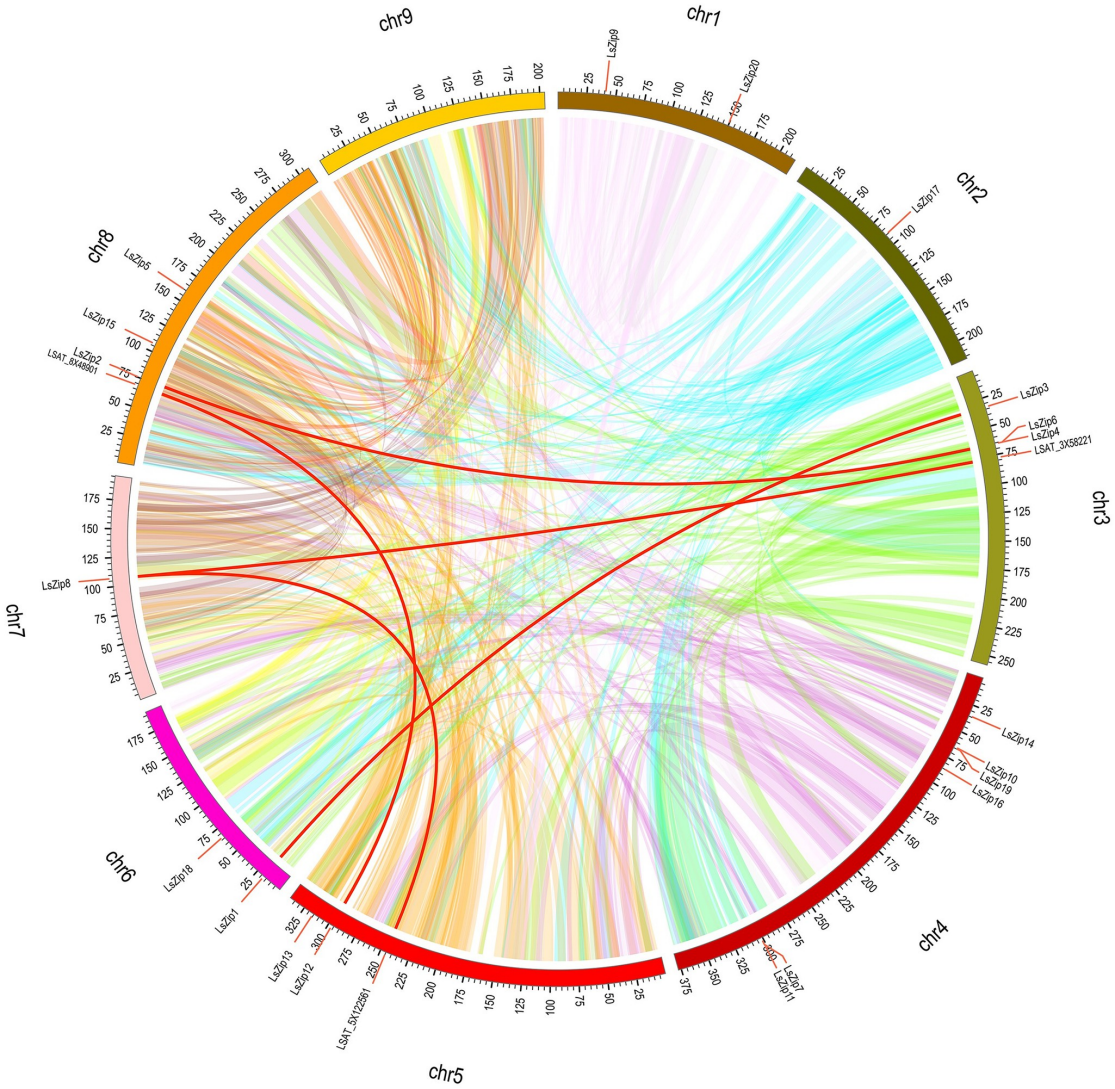
Schematic representations for chromosomal distribution and interchromosomal relationships of lettuce *ZIP* genes. Different colored lines indicate all synteny blocks in the lettuce genome. The red line indicates a gene pair that is duplicated with *LsZIP* genes. The chromosome number is indicated at the top of each chromosome. The black text indicates the gene name of the *LsZIP* family, and the gray text indicates the gene name that has a collinearity relationship with the *LsZIP* genes. The lengths of the chromosomes are marked using scales, with each small scale indicating 500,000 amino acids, and every 25th scale is marked using a number.

As shown in Fig 4, the *LsZIP* gene was distributed among the 8 chromosomes of lettuce. Chromosome 4 contains most the *ZIP* genes, while chromosome 9 contains no *LsZIP* genes. Chromosome1, chromosome3 and chromosome6 have two genes, chromosome3 and chromosome8 have three genes, chromosome2 and one gene in chromosome7. There was no positive correlation between chromosome length and the number of *ZIP* genes. After calculating the tandem replication events in the *LsZIP* gene family of lettuce by blastN, four tandem repeat genes (*LsZIP4/6* and *LsZIP7/11*) were found. Two pairs of tandem repeat genes are located on chromosomes 3 and 4. Although *LsZIP10* and *LsZIP19* are also located on chromosome 4 and the distance between them is very small, surprisingly there is no tandem replication event between them. In addition to the tandem repetitive events, MCScanX methods identified 5 segmental repetitive events, containing 4 *ZIP* genes on nine chromosomes of lettuce. Several repetitive segmental events did not occur in the *LsZIP* gene family, such as *LsZIP8*-*LAST_5X122561*/*LAST_3X58221*, *LsZIP12*- *LAST_8X48901*. The gene protein sequences of four segmented events overlapping with the lettuce *ZIP* gene family were submitted to HMMER for searching, and no specific domains were found. These results suggest that some *LsZIP* genes may have evolved by gene duplication and that fragment duplication events are one of the driving forces of *LsZIP* gene evolution.

**Fig 5:**
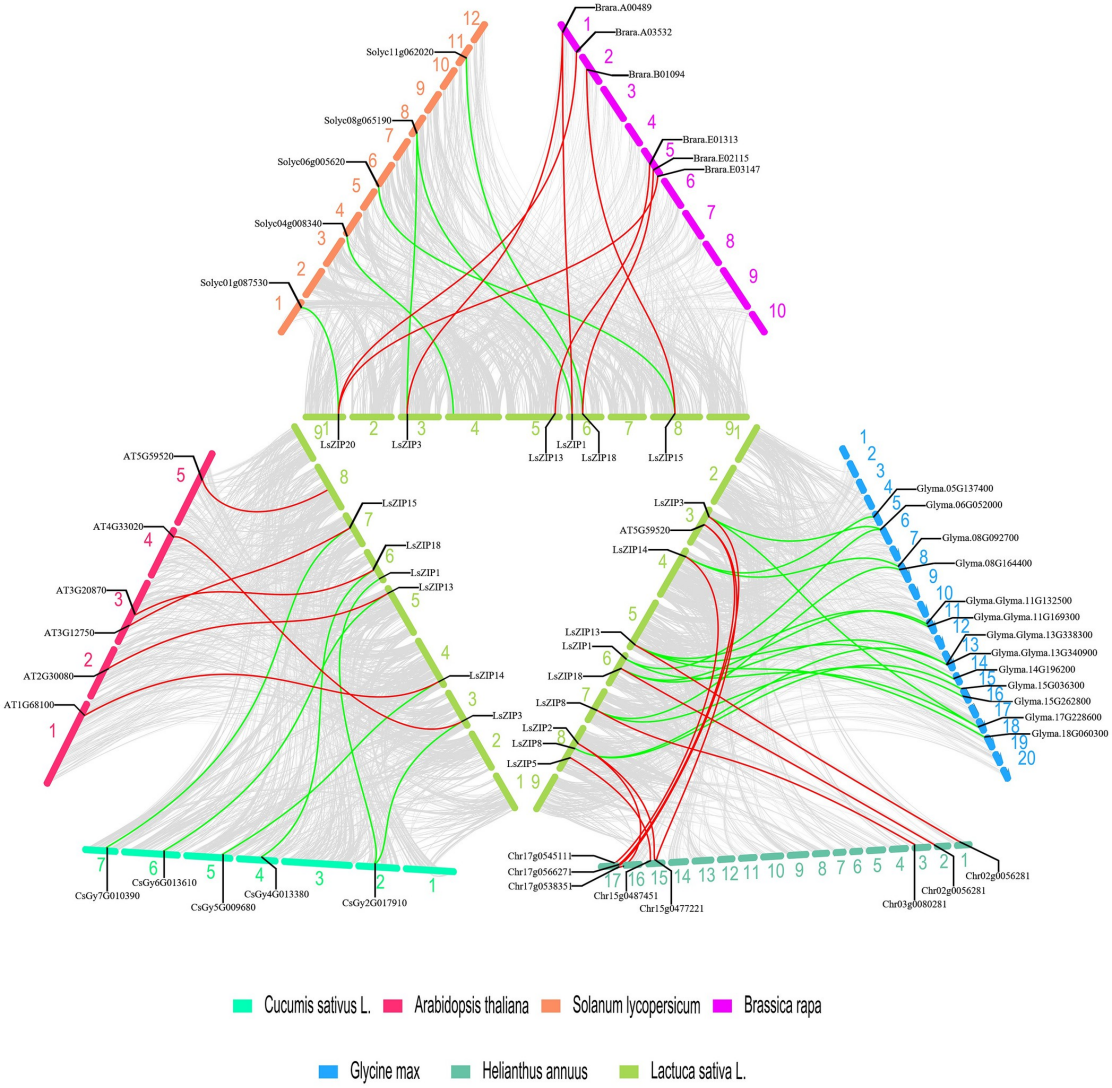
Synteny analysis of *ZIP* genes between lettuce and six different species. Different colors represent the chromosomes of different species, and the chromosome numbers are represented by numbers. Gray lines in the background indicate the collinear blocks within lettuce and other plant genomes, while the red and green lines highlight the syntenic *ZIP* gene pairs. The black text indicates the gene names of the *LsZIP* genes and the gene ID that has a synthesis relationship with the *LsZIP* genes.

**Fig 6:**
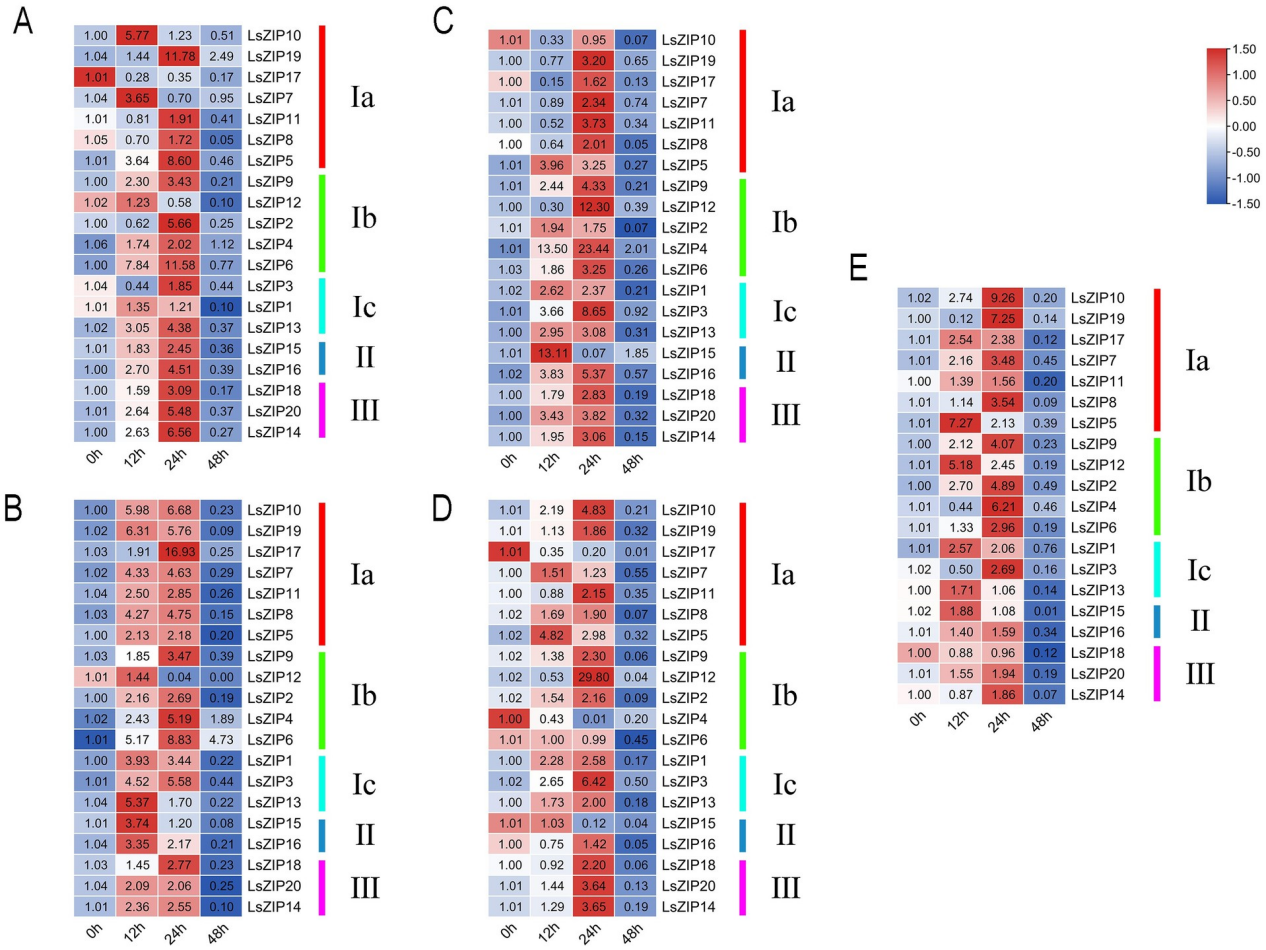
Heatmap representation of the expression levels of 20 *LsZIP*s among different treaments. Quantitative reverse transcription polymerase chain reaction (qRT-PCR) analyses were used to assess the transcript levels of *LsZIP*s in leaves sampled at 0 h, 12 h,24h and 48 h after different treatment in lettuce seedlings. Lower and higher levels of expression levels are indicated by red and blue, and the median level is indicated by white. The heatmap was constructed using TBtools. Where A: represents the relative expression of 20 *LsZIP* genes under Zn-excess treatment; B: represents the relative expression of 20 *LsZIP* genes under Zn-deficiency treatment; C: represents the relative expression of 20 *LsZIP* genes under Fe-excess treatment; D: represents the relative expression of 20 *LsZIP* genes under Fe-deficiency treatment; and E: Relative expression of 20 *LsZIP* genes in response to Cd stress.

To further infer the phylogenetic mechanisms of the lettuce *LsZIP* gene family, we constructed a comparative synonymous map of lettuce related to six different species, including Arabidopsis, cabbage, Cucumber, Tomato, Soybean and Sunflower. Monocotyledonous plants (such as rice, sorghum and maize) were not used. The results are shown in Figs 5 and 6. In terms of total number of gene pairs, lettuce and soybean have the most gene pairs (23,812), followed by sunflower (19,540), tomato (15,760), cabbage (14,544), Arabidopsis (13,136) and cucumber (12,384). Although soybean has far more orthologous gene pairs than sunflowers, they are very scattered on the chromosomes (Fig 6), whereas the sunflower, tomato and cucumber orthologous gene pairs are more concentrated. Regarding the *LsZIP* genes a syntenic relationship, 15 soybean genes have syntenic relationship with 7 *LsZIP* genes. In sunflower, 10 genes have a syntenic relationship with 8 *LsZIP* genes. In cabbage, 7 genes have shown syntenic relationship with 6 *LsZIP* genes. In tomato, cucumber and Arabidopsis, 6 genes have syntenic relationship with 6 *LsZIP* genes. The numbers of relationship with 6 *LsZIP* genes. Pairs between the other six species (soybean, Arabidopsis, tomato, cucumber, sunflower, cabbage) were 902, 569, 499, 450, 411 and 354. The *LsZIP3* and *LsZIP18* genes are syntenically relationship in all six species, suggesting that these orthologous pairs may share a common ancestor. Some *LsZIP* genes were found to be associated with two or three syntenic gene pairs (especially in soybean). These genes are thought to have played a crucial role in evolution. It is interesting to note the lettuce *LsZIP* gene occurs in most syntenic relationships with soybean and not with sunflower in the composites. However, sunflower has a syntenic relationship with most of the *ZIP* genes of lettuce. In addition, the *LsZIP1* gene has been identified as a collinear gene pair between lettuce and cucumber/soybean/tomato/cabbage but not in Arabidopsis/sunflower, and the *LsZIP2/LsZIP6* genes are unique collinear gene pairs of lettuce and sunflower. This may could indicate differences between these direct pairs and different species.

To better understand the evolutionary constraints acting on the *ZIP* gene family, the Ka/Ks ratio of all *ZIP* genes was calculated as shown as S3 Table. The Ka/Ks of all *ZIP* genes, including all segmental and tandem duplicated *ZIP* genes, are all less than 1, indicating that purifying selection has played an important role in the evolution of these synteny genes.

### Relative expression of 20 LsZIP genes under five element treatments

To confirm that they are involved in the transfer of elements and their expression in the transfer of elements, the expression of 20 genes from five groups under Zn-deficiency, Zn-excess, Fe-deficiency, Fe-excess and Cd stresses were validated by qRT—PCR. The results of qRT-PCR analysis showed that under the stress of different elements, 20 *LsZIP* genes were induced to different events. In the Zn-excess treatment, four *LsZIP* genes were most highly expressed at 12 h, fifteen *LsZIP* genes were most highly expressed at 24 h, and the relative expression of one *LsZIP* gene was lower than that of the control; in the Zn-deficiency treatment, six *LsZIP* genes were most highly expressed at 12 h, and fourteen *LsZIP* genes were most highly expressed at 24 h. In Fe-excess treatment, four *LsZIP* genes had the highest expression at 12h, fifteen *LsZIP* genes had the highest expression at 24h, and the relative expression of one *LsZIP* gene was lower than the control; in Zn-deficiency treatment, two *LsZIP* genes had the highest expression at 12h, and fourteen *LsZIP* genes had the highest expression at 24h. The relative expression of four *LsZIP* genes was lower than that of the control. It is worth noting that most of these *LsZIP* genes are up-regulated under each element stress, indicating that they are actively involved in element transport. At the same, *LsZIP* genes also vary in their response to different element stresses. For example, *LsZIP17* (Group Ia) was dramatically induced more than 16-fold under Zn-deficiency; *LsZIP19* (group Ia) was dramatically induced by more than 11-fold under Zn-excess, but under Fe-excess and Cd stress, the change in expression of *LsZIP17* was only 1.62-fold and 2.54-fold, respectively; *LsZIP19* still has 6.31-fold and 7.25-fold expression. Under Fe-excess stress, the expression of *LsZIP12* and *LsZIP4* (group Ib) was significantly up-regulated more than 12-fold and 23-fold, *LsZIP12* was also dramatically increased by 29-fold under Fe-deficiency, but the gene expression of *LsZIP4* was significantly down-regulated. Under Cd stress, the expression levels of *LsZIP12* and *LsZIP4* increased 5.18- fold and 6.21-fold, respectively. *LsZIP1*, *LsZIP3* and *LsZIP13* (group Ic) were upregulated under all element stresses. *LsZIP15* and *LsZIP16* (group II) were up-regulated under Zn-deficiency, Zn-excess and Fe-excess. However, the expression of *LsZIP15* was up-regulated 13.11-fold under Fe-excess stress. Under Fe-deficiency stress and Cd stress, the expression of the genes is slightly up-regulated. This indicates the important role of *LsZIP15* in the response to Fe-excess stress. *LsZIP14*, *LsZIP18* and *LsZIP20* (group III) showed similar expression patterns as *LsZIP15* and *LsZIP16*, but they have the highest gene expression under Zn stress, and they were actually not hypersensitive to Cd stress.

## Discussion

Previous studies have identified the *ZIP* gene family in plants such as Arabidopsis and rice. In addition to these genes playing a vital role in the transport of Zn and Fe, leaves are also involved in the transport of heavy metals such as Cd. However, there are no studies on the function and gene expression of *LsZIP* under elemental stress in lettuce. Therefore, this study aims to analyze the *LsZIP* gene family in the hope of investigating its possible effects on the transport of elements in lettuce and the accumulation of Cd. The whole genome sequence of lettuce allows researchers to identify and characterize different gene families in lettuce [29]. However, reports on *ZIP* genes are mainly focused on the transport and uptake of Zn and Fe, and there are few reports on the response to Cd, especially in leafy vegetables. As a leafy vegetable grown worldwide, lettuce has a high uptake capacity for Cd, but there are no reports on the identification of the *ZIP* gene in lettuce. In this study, based on previous research and the study of *LsZIP*s characteristics in other plants, we identified a total of 20 *LsZIP*s by genome-wide identification. Then, the gene structure, chemical and physical properties, phylogenetic relationships, conservative motifs, cis-regulatory element composition, chromosome location, synlinearity analysis and expression patterns under different element stresses were studied. The results provide valuable and important information to better explore the function and evolutionary relationship of these *LsZIPs*.

In our study, a total of 20 *ZIP* gene members were identified by searching the lettuce genome. According to the Evalue obtained by hmmsearch, these members are named *LsZIP1* to *LsZIP20*. The *LsZIP* genes usually have 1–3 exons, and the number of exons in the same subfamily is very similar. The *LsZIP* gene in the group III contains the largest number of exons. The *LsZIP* genes in group Ib and group II have conservative gene structure, suggesting that are closely related evolutionarily. Interestingly, the *LsZIP* gene in group III contains abundant short exons. Xu [47] hypothesizes that plants acquire more introns in the early stages of evolution because the rate of intron loss is often greater than the rate of intron acquisition when fragments are duplicated. Therefore, the genes in group III may be relatively primitive in the *LsZIP* gene family.

According to chromosomal mapping analysis, *LsZIP* genes are distributed on chromosomes 1–8 of lettuce, whereas chromosome 9 does not contain *LsZIP* genes. In addition, the six *LsZIP* genes were distributed on chromosome 4, more than on the other chromosomes. The appearance of gene family members is usually accompanied by gene duplication and leads to new functions in the evolution of plant genomes. In our study, only two pairs of *LsZIP* genes were identified as segmental duplications. However, in *LsZIP8* and *LsZIP12*, segmental duplications were identified as genes outside the *LsZIP* gene family, even if they did not contain specific domains. Among them, *LsZIP*8 was identified as segmental duplications with two genes. Syntenic *LsZIP*s were located at the beginning or end of chromosomes. In addition, there were three pairs of *LsZIP* genes on chromosomes, which are very close and closely related to each other. This implies that they may have a similar origin. Therefore, to further study the evolutionary relationship of this gene family, it is of great important to study their repetitive relationship.

After further analysis of the motifs, it was revealed that there are very similar motifs in groups I and II and their subgroups, which indicates that they are very conservative in evolution. No similar motif was found in group III. The *LsZIP* gene family contains cis-acting elements that respond to hormones and stress, such as methyl jasmonate, abscisic acid, auxin, and salicylic acid. In a previous study, Akther [48] found that tolerance to Zn deficiency in tomato is dependent on auxin signaling; Song [49] found that ABA can reduce Zn uptake and accumulation in grapes by inducing *ZIP* gene expression; furthermore, Kabir [50] found that salicylate and methyl jasmonate response elements in three *ZIP* genes of sunflower were regulated in response to iron deficiency; Matsuoka [51] found that erythromycin positively regulated *AtZIP1* expression under iron sufficiency or iron deficiency conditions. This suggests that *LsZIP* gene expression may be induced by several plant hormones, such as salicylic acid, MeJA, abscisic acid, gibberellin, and auxin. Methyl jasmonate is an important hormone in plants. In the presence of insect pests, methyl jasmonate can serve as a key signaling factor of plant resistance to biological invasion [52]. In addition, methyl jasmonate can also defend against biological and abiotic stresses by triggering defense mechanisms and regulating growth [53]. Auxin is a hormone that controls plant development, while tryptophan is a key amino acid in the synthesis of auxin indoleacetic acid, and Zn is involved in the synthesis of tryptophan, so it can indirectly regulate plant growth and development through auxin [54]. Similar to auxin, gibberellin can also regulate a number of plant developmental processes, such as seed germination and fruit development [55]. Abscisic acid not only regulates plant senescence, but abscisic acid can also control plant transpiration by regulating the stomatal conductance [56], thereby affecting the absorption of elements. Salicylic acid (SA), as an important signaling molecule, can improve plant resistance to stress and regulate plant growth [57]. In addition, a variety of cis-elements related to defense and stress response were found in the promoter region of the *LsZIP* gene. This suggests that the *LsZIP* gene family may regulate plant growth through hormone signaling pathways to adapt to elemental stress.

The *ZIP* family is not only responsible for the transport of Zn and Fe in plants, but also other divalent metal ions, including some heavy metal ions. For example, *BcZIP2* can transport Zn^2+^, Fe^2+^, Cd^2+^ and Mn^2+^, and under Hydrogen-rich water treatment, the relative expression levels of *BcZIP2* and *BcZI42* are significantly down-regulated, and the Cd content in pakchoi is also reduced [17]. After using functional complementation of yeast mutants to correlate specific regulation by metals with transport activity in Arabidopsis thaliana, *AtZIP2* and *AtZIP4* were identified as involved in copper transport [16]. Under Cd stress, the expression of the *SlZIP4* gene in tomato increased, and shwed a concentration-dependent increase with increasing Cd concentration. A similar situation was also observed in rice, where the relative expression of the *OsZIP4* was increased gene under Cd stress [21]. In this work, we analyzed the expression level of *LsZIP* gene in leaves of lettuce treated with Zn-deficiency, Zn-excess, Fe-deficiency, Fe-excess and Cd for 12, 24, and 48 hours. Our results showed that most of the 20 *LsZIP*s were up-regulated under Zn treatment, Fe treatment and Cd stress, *LsZIP* genes were mainly expressed at 12 h and 24 h. Most of the *LsZIP* genes reached their highest expression level at 24 h and responded most strongly to elemental stress, but the extent of the up-regulation was differed among the different stress treatments. For example, *LsZIP4* was significantly up-regulated 23.44-folds under Fe-excess treated, but gene expression was significantly down-regulated under Fe-deficient treatment. *LsZIP12* was significantly up-regulated 12.3-fold and 29.8-fold under Fe-excess and Fe-deficient treatment. But not sensitive to Zn treatment. The same phenomenon also occurred in *LsZIP17*. Its gene expression was significantly down-regulated under Zn-excess and Fe-deficient treatments, but it was significantly up-regulated by 16.93-fold in Zn-deficiency. This indicates that different *LsZIP*s have different roles in the transport of elements in lettuce and show a tendency toward the elements. Interestingly, the relative expression level of many *LsZIP* genes increased under Cd treatment. For example, the expression level of *LsZIP10* increased by 9.26-flods. Compared with the group I, the expression levels of group II and group III under Cd stress increased less. Although there are reports of the involvement of *ZIP* genes in Cd transport in Arabidopsis and tobacco [22, 58], the number of *LsZIP* genes in lettuce in response to Cd stress is far greater than these, which may explain why lettuce has a high accumulation and transport capacity for Cd.

In previous studies, the *ZIP* gene family was mainly focused on the transport of Zn and Fe [4, 10] and less attention was paid to the transport of other metal ions, especially Cd. Zn is beneficial for growth, while Cd is a dangerous heavy metal that can inhibit plant growth only at low concentration. In previous studies, researchers have found that Cd stress can reduce the absorption of Zn by plants [59], while the appropriate addition of Zn can reduce the absorption of Cd by plants and improve the utilization of other elements [60], but studies have also found that Zn The inhibition of Cd absorption depends on the ratio of the two concentrations [61]. The *ZIP* gene family may play a crucial role in regulating the transport of Zn and Cd. For example, in rice, *OsZIP1* can excrete Zn and Cd to prevent excessive accumulation [62]. Studies in tobacco revealed that Cd can transport Zn by stimulating the expression of *ZIP* genes [22]. Lettuce is one of the important dietary sources for Cd intake. It is very important to find related genes that can reduce the uptake of Cd by lettuce.

## Conclusions

In the current study, we performed an analysis of 20 *ZIP* genes in the lettuce genome. We investigated their physiochemical properties, cis-regulatory elements, motif composition, gene structures and phylogenetic relationships. Collinearity analysis and chromosomal mapping revealed the duplication relationship of lettuce *LsZIPs*. These *LsZIPs* were divided into three groups distributed on 8 chromosomes and comprising 1–7 exons. Phylogenetic analysis showed that the *ZIP* proteins of lettuce were divided into three types and five *LsZIP* genes were closely related to monocots. Synonymous analysis showed that two *LsZIP* genes were duplicated by segmental. The *LsZIP* gene was found to have a variety of cis-acting elements related to hormones and anti-stress response factors. Finally, expression analysis showed that the relative expression of the *ZIP* gene after treatment was generally higher at 12h and 24h, and decreased at 48h. The relative expression of 18 *ZIP* genes increased significantly with Cd stress. Overall, this study provides insights into the potential functional role of the *ZIP* gene in lettuce. Comprehensive analysis will help select candidate *LsZIP* genes for further functional characterization. In the next study, three candidate genes (*LsZIP4*, *LsZIP10*, and *LsZIP19*) with high expression under Cd stress will be selected to further verify their functions.

## Supporting information

S1 Fig Multiple sequence alignment of the predicted 20 lettuce *ZIP* proteins with the other 113 ZIP proteins. Identical amino acids are indicated with red shading (100%) and similar amino acids are indicated with yellow (80%) and green (60%) shading. The seven domains are shown as a red line above the sequences.
(TIF)

S1 Table The sequences of primers used for qRT-PCR. (DOCX)

S2 Table List of *ZIP* genes in lettuce. Note: a: Name of gene model was modified from the annotation of the lettuce genome v7 (from NCBI), the prefix ‘*Ls*’ indicating the lettuce species abbreviated from *L*. *sativa*; b: NCBI database unique digital identifier for the gene; c: Lo-cations represent the coding region of the gene. can estimate of the stability of your protein in a test tube, a protein whose instability index is smaller than 40 is predicted as stable, a value above 40 predicts that the protein may be unstable.
(DOCX)

S3 Table KA/KS of 20 *LsZIP* genes. (DOCX)

S4 Table Predicted secondary structure of lettuce *ZIP* protein. (DOCX)
