# Supplementary figures and images for: Genome-wide identification of the ZIP gene family in lettuce (Lactuca sativa L.) and expression analysis under different element stress
Source: PLoS One. 2022 Sep 28;17(9):e0274319. doi: 10.1371/journal.pone.0274319 (PMC9518877; doi:10.1371/journal.pone.0274319)

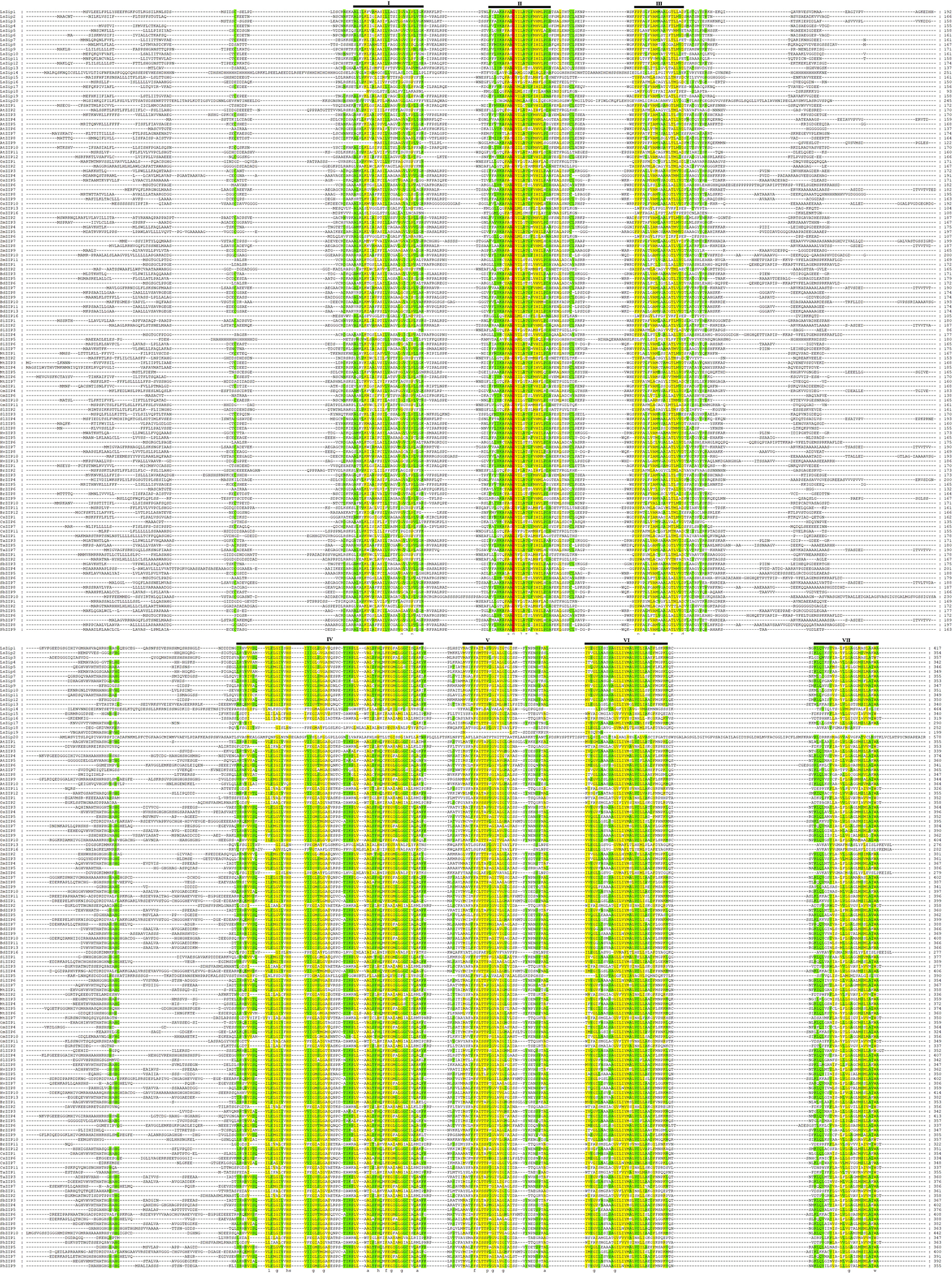

Supplement: S1 Fig — Identical amino acids are indicated with red shading (100%) and similar amino acids are indicated with yellow (80%) and green (60%) shading. The seven domains are shown as a red line above the sequences. (TIF) [file pone.0274319.s001.tif]
