# Supplementary material for: Genome-wide identification of the ZIP gene family in lettuce (Lactuca sativa L.) and expression analysis under different element stress
Source: PLoS One. 2022 Sep 28;17(9):e0274319. doi: 10.1371/journal.pone.0274319 (PMC9518877; doi:10.1371/journal.pone.0274319)
Supplement: S1 Table — (DOCX) [file pone.0274319.s002.docx]

**Table S2. The sequences of primers used for qRT-PCR**

| Gene Name | Forward Primer Sequence (5’ -3’) | Reverse Primer Sequence (5’ -3’ ) |
| --- | --- | --- |
| *LsZIP1* | AGCCAAATTCGGGAGCCTTCATTC | GTTCCAATTCCGACACCCACAGG |
| *LsZIP2* | TCCCTCTTGTCACCCGCTCTG | AACTGGACCTCAACATGCCGAAAG |
| *LsZIP3* | TGACCACACTGCTTGCTGACTTC | CCAACGGAACGACCACGGATG |
| *LsZIP4* | ACATGGCGTTGGTCGATCTTCTTG | GACGGCGGCGAAAGATTTGATTTG |
| *LsZIP5* | GTGGCGGCTGTGGTGGTTATG | ACCCTCCTGCTTCTCTTCATCTCC |
| *LsZIP6* | TCATCGGCATCGCTACAATCCTTG | TGGGCTAAGGGCAGGGATAGAAC |
| *LsZIP7* | ACATGCGAACGAGAAGAAGACGAG | ACACCGATGGCACCAAATATGAGG |
| *LsZIP8* | CACCACCACCACAAGCGACAG | GGATGCTCACACCAATTCCACCAG |
| *LsZIP9* | CACCTCCTCATCCTCTCACTCCTC | TGGGTGGTTGGGTGGTAGAGTTAG |
| *LsZIP10* | CCTAATCATCACCGTAGCGTCTGC | TTGCCGACAAAGGGTAGAGAAACG |
| *LsZIP11* | TTAGCCGACTGCACTTGCGAAC | ACACCGATGCCACCAAATATGAGG |
| *LsZIP12* | CCTGCTCTCCACCTTCACAACAC | GCAGCAATGGCGATGACTTTAAGC |
| *LsZIP13* | CGCCGTAGACTCCGCCAGAG | CCGATATTCCGACGACGCTTGTG |
| *LsZIP14* | CTGTGACCACTCGCATGACCATC | TCCTCTTCCGCCAGTTCTTCCG |
| *LsZIP15* | GCATCGGCTGGTTACCTTCTCAC | ACCACCACCATGACCTCCTCTTC |
| *LsZIP16* | CGCCTTCGCCTTTGGAATTTCAAG | ATCTGCGACCCGACCCTGTG |
| *LsZIP17* | CGGTAGCAGTTGAAGGGGTTGAC | ATTGGCGTGAGAATGGTGATGGG |
| *LsZIP18* | AACGGTTTCTGGGCTTGCTGAG | GGCCATAACTCCACCAACTGCTC |
| *LsZIP19* | CTGGGTTTGTGGCGATGGTA | AGCCGTCTGAGCCCTAACTTGG |
| *LsZIP20* | GGTCCATTACTAGGCGGCAACATC | CCAGGTGGCGAGGAGGAAGG |
| *action* | CCGTGAACCCAAACCCTGATGAG | CGCATCCGCCTTCCTTAGTAACTC |
