# Supplementary material for: Genome-wide identification of the ZIP gene family in lettuce (Lactuca sativa L.) and expression analysis under different element stress
Source: PLoS One. 2022 Sep 28;17(9):e0274319. doi: 10.1371/journal.pone.0274319 (PMC9518877; doi:10.1371/journal.pone.0274319)
Supplement: S2 Table — Note: a: Name of gene model was modified from the annotation of the lettuce genome v7 (from NCBI), the prefix ‘Ls’ indicating the lettuce species abbreviated from L. sativa; b: NCBI database unique digital identifier for the gene; c: Lo-cations represent the coding region of the gene. can estimate of the stability of your protein in a test tube, a protein whose instability index is smaller than 40 is predicted as stable, a value above 40 predicts that the protein may be unstable. (DOCX) [file pone.0274319.s003.docx]

**Table S1.** List of *ZIP* genes in lettuce

| ^a^Gene Name | ^b^Gene ID | ^c^Location in Chromosome  (Chr: start - end) | Protein Subcellular Localization Prediction | Deduced peptides | | | | Evalve |
| --- | --- | --- | --- | --- | --- | --- | --- | --- |
|  |  |  |  | Protein.Length  (aa) | MW (kDa) | Theoretical pI | ^c^Instability index |  |
| *LsZIP1* | 111906062 | 6:17789745-17792393 | Plasma membrane | 417 | 44.63 | 6.24 | 36.25 | 3.20E-76 |
| *LsZIP2* | 111896160 | 8:75470642-75472755 | Plasma membrane | 354 | 37.74 | 8.92 | 34.18 | 8.70E-74 |
| *LsZIP3* | 111917238 | 3:33130126-33132674 | Plasma membrane | 357 | 37.86 | 5.4 | 38.02 | 8.60E-73 |
| *LsZIP4* | 111891111 | 3:65632264-65633774 | Plasma membrane | 342 | 36.22 | 8.58 | 34.14 | 1.50E-72 |
| *LsZIP5* | 111893633 | 8:160058403-160059977 | Plasma membrane | 346 | 37.15 | 6.03 | 31.95 | 4.60E-71 |
| *LsZIP6* | 111891133 | 3:65659735-65661118 | Plasma membrane | 349 | 36.8 | 8.73 | 34.15 | 9.20E-71 |
| *LsZIP7* | 111901856 | 4:301335083-301338807 | Plasma membrane | 355 | 38.19 | 6.05 | 27.18 | 3.10E-70 |
| *LsZIP8* | 111897073 | 7:107007769-107009628 | Plasma membrane | 353 | 37.68 | 6.41 | 33.6 | 2.40E-69 |
| *LsZIP9* | 111900088 | 1:40972757-40974027 | Plasma membrane | 347 | 37.38 | 8.88 | 39.75 | 1.50E-68 |
| *LsZIP10* | 111891828 | 4:64006738-64008355 | Plasma membrane | 351 | 37.73 | 6.3 | 26.72 | 1.80E-68 |
| *LsZIP11* | 111901882 | 4:301193570-301195875 | Plasma membrane | 349 | 37.51 | 6.25 | 29.19 | 2.40E-67 |
| *LsZIP12* | 111878341 | 5:296864274-296865646 | Plasma membrane | 350 | 37.83 | 8.54 | 43.12 | 3.80E-67 |
| *LsZIP13* | 111912685 | 5:315727073-315728832 | Plasma membrane | 340 | 36.71 | 6.04 | 33.86 | 1.40E-65 |
| *LsZIP14* | 111891970 | 4:34471791-34474446 | Vacuolar | 464 | 51.04 | 5.79 | 41.48 | 1.50E-55 |
| *LsZIP15* | 111880346 | 8:107012832-107014771 | Plasma membrane | 340 | 36.92 | 6.25 | 32.39 | 2.90E-51 |
| *LsZIP16* | 111887358 | 4:84936620-84937984 | Plasma membrane | 319 | 34.4 | 5.31 | 24.06 | 4.50E-48 |
| *LsZIP17* | 111894361 | 2:89572291-89574361 | Plasma membrane | 290 | 31.57 | 6.44 | 33.07 | 3.70E-41 |
| *LsZIP18* | 111877483 | 6:66610663-66614289 | Plasma membrane | 252 | 26.8 | 9.4 | 36.55 | 4.00E-22 |
| *LsZIP19* | 111896002 | 4:64768669 -64769283 | Vacuolar | 199 | 21.48 | 5.7 | 30.02 | 3.80E-19 |
| *LsZIP20* | 111908302 | 1:149740337-149742568 | Plasma membrane | 578 | 60.51 | 6.49 | 32.21 | 9.90E-12 |

Note: a: Name of gene model was modified from the annotation of the lettuce genome v7 (from NCBI), the prefix ‘*Ls*’ indicating the lettuce species abbreviated from *L. sativa*; b: NCBI database unique digital identifier for the gene; c: Lo-cations represent the coding region of the gene. can estimate of the stability of your protein in a test tube, a protein whose instability index is smaller than 40 is predicted as stable, a value above 40 predicts that the protein may be unstable.
