# Supplementary material for: Genome-wide identification of the ZIP gene family in lettuce (Lactuca sativa L.) and expression analysis under different element stress
Source: PLoS One. 2022 Sep 28;17(9):e0274319. doi: 10.1371/journal.pone.0274319 (PMC9518877; doi:10.1371/journal.pone.0274319)
Supplement: S3 Table — (DOCX) [file pone.0274319.s004.docx]

**Table S3.** KA/KS of 20 *LsZIP* genes

| Seq_1 | Seq_2 | Ka | Ks | Ka_Ks |
| --- | --- | --- | --- | --- |
| LsZip2 | LsZip4 | 0.114618 | 1.32419 | 0.086557 |
| LsZip1 | LsZip3 | 0.191169 | 0.986668 | 0.193752 |
| LsZip4 | LsZip6 | 0.028774 | 0.187907 | 0.15313 |
| LsZip7 | LsZip11 | 0.102355 | 0.332154 | 0.308155 |
| LsZip1 | LsZip2 | 0.498283 | 2.282406 | 0.218315 |
| LsZip1 | LsZip5 | 0.499124 | 2.126631 | 0.234702 |
| LsZip1 | LsZip12 | 0.505227 | 2.417126 | 0.20902 |
| LsZip1 | LsZip13 | 0.588603 | 2.893967 | 0.20339 |
| LsZip1 | LsZip14 | 1.183492 | 3.250396 | 0.364107 |
| LsZip1 | LsZip15 | 0.928965 | 2.692072 | 0.345075 |
| LsZip1 | LsZip16 | 0.935274 | 2.668027 | 0.350549 |
| LsZip2 | LsZip3 | 0.546149 | 2.481864 | 0.220056 |
| LsZip2 | LsZip6 | 0.114286 | 1.248969 | 0.091505 |
| LsZip2 | LsZip7 | 0.454685 | 3.111833 | 0.146115 |
| LsZip2 | LsZip10 | 0.455609 | 1.789385 | 0.254618 |
| LsZip2 | LsZip11 | 0.481729 | 3.437985 | 0.14012 |
| LsZip2 | LsZip12 | 0.322424 | 3.436393 | 0.093826 |
| LsZip2 | LsZip13 | 0.628214 | 2.996828 | 0.209626 |
| LsZip2 | LsZip15 | 0.922143 | 2.279178 | 0.404595 |
| LsZip2 | LsZip17 | 0.530649 | 2.252312 | 0.235602 |
| LsZip2 | LsZip19 | 0.594182 | 1.431051 | 0.415207 |
| LsZip3 | LsZip4 | 0.574194 | 2.716288 | 0.211389 |
| LsZip3 | LsZip5 | 0.509904 | 2.93492 | 0.173737 |
| LsZip3 | LsZip8 | 0.447072 | 3.589576 | 0.124547 |
| LsZip3 | LsZip9 | 0.533833 | 2.483843 | 0.214922 |
| LsZip3 | LsZip11 | 0.548749 | 2.698028 | 0.203389 |
| LsZip3 | LsZip12 | 0.50542 | 2.450145 | 0.206282 |
| LsZip3 | LsZip13 | 0.595935 | 2.275144 | 0.261933 |
| LsZip3 | LsZip14 | 1.15074 | 3.068672 | 0.374996 |
| LsZip3 | LsZip15 | 0.823959 | 2.444725 | 0.337035 |
| LsZip3 | LsZip16 | 0.906408 | 5.668888 | 0.159892 |
| LsZip3 | LsZip19 | 0.630735 | 2.975765 | 0.211957 |
| LsZip4 | LsZip9 | 0.309772 | 2.108127 | 0.146942 |
| LsZip4 | LsZip10 | 0.515096 | 2.080608 | 0.24757 |
| LsZip4 | LsZip11 | 0.484502 | 4.74125 | 0.102189 |
| LsZip4 | LsZip13 | 0.645439 | 2.351443 | 0.274486 |
| LsZip4 | LsZip16 | 0.932575 | 2.448162 | 0.380929 |
| LsZip4 | LsZip17 | 0.560531 | 3.514392 | 0.159496 |
| LsZip5 | LsZip7 | 0.391248 | 1.913195 | 0.2045 |
| LsZip5 | LsZip9 | 0.449604 | 3.354691 | 0.134022 |
| LsZip5 | LsZip10 | 0.42708 | 1.84199 | 0.231858 |
| LsZip5 | LsZip11 | 0.415891 | 2.101233 | 0.197927 |
| LsZip5 | LsZip14 | 0.926275 | 3.716036 | 0.249264 |
| LsZip5 | LsZip15 | 0.877214 | 3.364299 | 0.260742 |
| LsZip5 | LsZip16 | 0.881737 | 5.274846 | 0.167159 |
| LsZip5 | LsZip18 | 0.875159 | 6.02183 | 0.145331 |
| LsZip5 | LsZip19 | 0.568988 | 1.22387 | 0.464909 |
| LsZip6 | LsZip9 | 0.31221 | 1.999847 | 0.156117 |
| LsZip6 | LsZip10 | 0.460432 | 1.80208 | 0.2555 |
| LsZip6 | LsZip13 | 0.636044 | 1.916125 | 0.331943 |
| LsZip6 | LsZip16 | 0.960965 | 2.371211 | 0.405263 |
| LsZip6 | LsZip17 | 0.54831 | 1.99612 | 0.274688 |
| LsZip6 | LsZip18 | 0.890329 | 2.911292 | 0.305819 |
| LsZip6 | LsZip19 | 0.623955 | 3.378032 | 0.18471 |
| LsZip7 | LsZip10 | 0.204646 | 1.143817 | 0.178915 |
| LsZip7 | LsZip14 | 1.085947 | 2.027873 | 0.535511 |
| LsZip7 | LsZip15 | 1.08332 | 2.452778 | 0.441671 |
| LsZip7 | LsZip16 | 0.859838 | 1.993201 | 0.431386 |
| LsZip7 | LsZip17 | 0.23861 | 2.046534 | 0.116592 |
| LsZip7 | LsZip19 | 0.250154 | 1.461528 | 0.171159 |
| LsZip9 | LsZip11 | 0.482872 | 3.035358 | 0.159082 |
| LsZip9 | LsZip12 | 0.043324 | 0.395374 | 0.109577 |
| LsZip9 | LsZip15 | 0.902402 | 3.271793 | 0.275813 |
| LsZip9 | LsZip17 | 0.550619 | 3.307115 | 0.166495 |
| LsZip10 | LsZip11 | 0.205605 | 1.41075 | 0.145742 |
| LsZip10 | LsZip13 | 0.598809 | 4.993683 | 0.119913 |
| LsZip10 | LsZip16 | 0.866919 | 4.158789 | 0.208455 |
| LsZip10 | LsZip17 | 0.162852 | 0.679688 | 0.239598 |
| LsZip10 | LsZip19 | 0.158999 | 0.472446 | 0.336545 |
| LsZip10 | LsZip20 | 0.960881 | 2.760383 | 0.348097 |
| LsZip11 | LsZip14 | 1.044232 | 3.102574 | 0.33657 |
| LsZip11 | LsZip15 | 1.024799 | 1.984211 | 0.516477 |
| LsZip11 | LsZip16 | 0.849805 | 2.110433 | 0.402669 |
| LsZip11 | LsZip17 | 0.250022 | 2.207303 | 0.11327 |
| LsZip11 | LsZip18 | 0.856004 | 1.905535 | 0.44922 |
| LsZip11 | LsZip19 | 0.284821 | 1.785814 | 0.159491 |
| LsZip12 | LsZip14 | 1.149814 | 3.263693 | 0.352305 |
| LsZip12 | LsZip15 | 0.950571 | 3.771777 | 0.252022 |
| LsZip12 | LsZip16 | 0.910122 | 4.634464 | 0.196381 |
| LsZip12 | LsZip17 | 0.584524 | 2.345558 | 0.249205 |
| LsZip13 | LsZip15 | 0.837989 | 2.813416 | 0.297855 |
| LsZip13 | LsZip16 | 0.912096 | 2.877955 | 0.316925 |
| LsZip13 | LsZip17 | 0.709093 | 2.432892 | 0.291461 |
| LsZip13 | LsZip19 | 0.83537 | 2.51791 | 0.331771 |
| LsZip14 | LsZip16 | 1.047412 | 1.895466 | 0.552588 |
| LsZip14 | LsZip18 | 1.139742 | 1.611531 | 0.707242 |
| LsZip15 | LsZip16 | 0.292373 | 2.07209 | 0.1411 |
| LsZip15 | LsZip17 | 0.848602 | 3.269849 | 0.259523 |
| LsZip15 | LsZip18 | 1.100243 | 2.466254 | 0.446119 |
| LsZip15 | LsZip20 | 0.936797 | 2.969732 | 0.315448 |
| LsZip16 | LsZip17 | 0.811223 | 2.165454 | 0.37462 |
| LsZip16 | LsZip20 | 0.827007 | 3.653667 | 0.22635 |
| LsZip17 | LsZip19 | 0.195509 | 0.940999 | 0.207767 |
| LsZip18 | LsZip19 | 1.000204 | 4.588903 | 0.217962 |
| LsZip18 | LsZip20 | 0.758497 | 1.735642 | 0.437012 |
| LsZip19 | LsZip20 | 0.780153 | 2.745024 | 0.284206 |
