# Supplementary material for: Genome-wide identification of the ZIP gene family in lettuce (Lactuca sativa L.) and expression analysis under different element stress
Source: PLoS One. 2022 Sep 28;17(9):e0274319. doi: 10.1371/journal.pone.0274319 (PMC9518877; doi:10.1371/journal.pone.0274319)
Supplement: S4 Table — (DOCX) [file pone.0274319.s005.docx]

**S4 Table. Predicted secondary structure of lettuce *ZIP* protein**

| Protein Name | Alpha helix (%) | Beta turn (%) | Random coil (%) | Extended strand (%) |
| --- | --- | --- | --- | --- |
| *LsZIP1* | 45.8 | 3.6 | 39.57 | 11.03 |
| *LsZIP2* | 51.98 | 4.24 | 30.79 | 12.99 |
| *LsZIP3* | 47.9 | 2.24 | 38.66 | 11.2 |
| *LsZIP4* | 49.12 | 1.75 | 35.96 | 13.16 |
| *LsZIP5* | 46.82 | 3.76 | 35.84 | 13.58 |
| *LsZIP6* | 46.42 | 2.87 | 36.1 | 14.61 |
| *LsZIP7* | 46.2 | 3.66 | 35.21 | 14.93 |
| *LsZIP8* | 47.03 | 2.55 | 36.54 | 13.88 |
| *LsZIP9* | 51.87 | 2.02 | 10.95 | 35.16 |
| *LsZIP10* | 47.58 | 2.85 | 13.11 | 36.47 |
| *LsZIP11* | 47.56 | 3.44 | 13.18 | 35.82 |
| *LsZIP12* | 51.72 | 4.7 | 29.15 | 14.42 |
| *LsZIP13* | 47.06 | 4.12 | 12.06 | 36.76 |
| *LsZIP14* | 40.09 | 5.17 | 44.83 | 9.91 |
| *LsZIP15* | 40.29 | 7.35 | 29.12 | 23.24 |
| *LsZIP16* | 48.57 | 3.43 | 35.14 | 12.86 |
| *LsZIP17* | 41.72 | 4.83 | 37.24 | 16.21 |
| *LsZIP18* | 59.92 | 4.37 | 23.81 | 11.9 |
| *LsZIP19* | 37.19 | 3.52 | 48.24 | 11.06 |
| *LsZIP20* | 44.46 | 7.44 | 26.47 | 21.63 |
